# Differential Diagnosis of a Canalicular Adenoma: A Case Report and Literature Review

**DOI:** 10.7759/cureus.78166

**Published:** 2025-01-28

**Authors:** Vasileios Papanikos, Olga Kouroukli, Vasileios Kakouris, Panayotis Dais

**Affiliations:** 1 Department of Otorhinolaryngology - Head and Neck Surgery, University General Hospital of Patras, Patras, GRC; 2 Department of Pathology, Evangelismos General Hospital of Athens, Athens, GRC; 3 Department of Microbiology, University General Hospital of Patras, Patras, GRC; 4 Department of Oral and Maxillofacial Surgery, Bradford Teaching Hospitals NHS Foundation Trust, Bradford, GBR

**Keywords:** canalicular adenoma (ca), clinical features, differential diagnosis, histopathology, lip lesions

## Abstract

We report the case of a 68-year-old female patient who was referred to our hospital for a painless, immobile, hard and well-circumscribed nodule of the upper lip that had been present for a decade. Surgical excision under local anaesthesia was the treatment of choice. After histological examination, a diagnosis of a canalicular adenoma, an uncommon benign salivary gland tumour, was made. No late recurrences occurred during a two-year follow-up period. Despite the broad clinical differential diagnosis, this tumour has a characteristic appearance under the microscope. Histological examination is indispensable for diagnosis and for excluding malignancy.

## Introduction

A canalicular adenoma (CA) is an uncommon, benign, epithelial neoplasm found throughout the oral cavity, originating mainly in minor salivary glands [1]. It was initially considered a variant of basal cell adenoma, but in 1991, the World Health Organization (WHO) classified it as a distinct tumour of the salivary glands. CAs represent approximately 1-3% of all salivary gland tumours and 4-21% of benign neoplasms in minor salivary glands [2,3]. They predominantly occur in the elderly, between the fifth and seventh decades of life, with a higher prevalence in women rather than in men, with a ratio of 1.8:1 [4]. Geographic and racial factors may influence the incidence of CAs. The most commonly reported location of CAs is the upper lip (80%), followed by buccal mucosa, hard palate, tongue and floor of the mouth [5]. Clinically, these tumours mainly appear as a painless, mobile, slowly growing, multifocal (13%) or solitary (87%), mucosal or submucosal swelling, which is mobile or firm on palpation [1,4]. Diagnosis is based on microscopic examination, which reveals the unique morphology of the tumour, confirmed by immunohistochemical analysis. The differential diagnosis includes other benign salivary gland tumours and importantly malignant tumours.

## Case presentation

**Figure 1:**
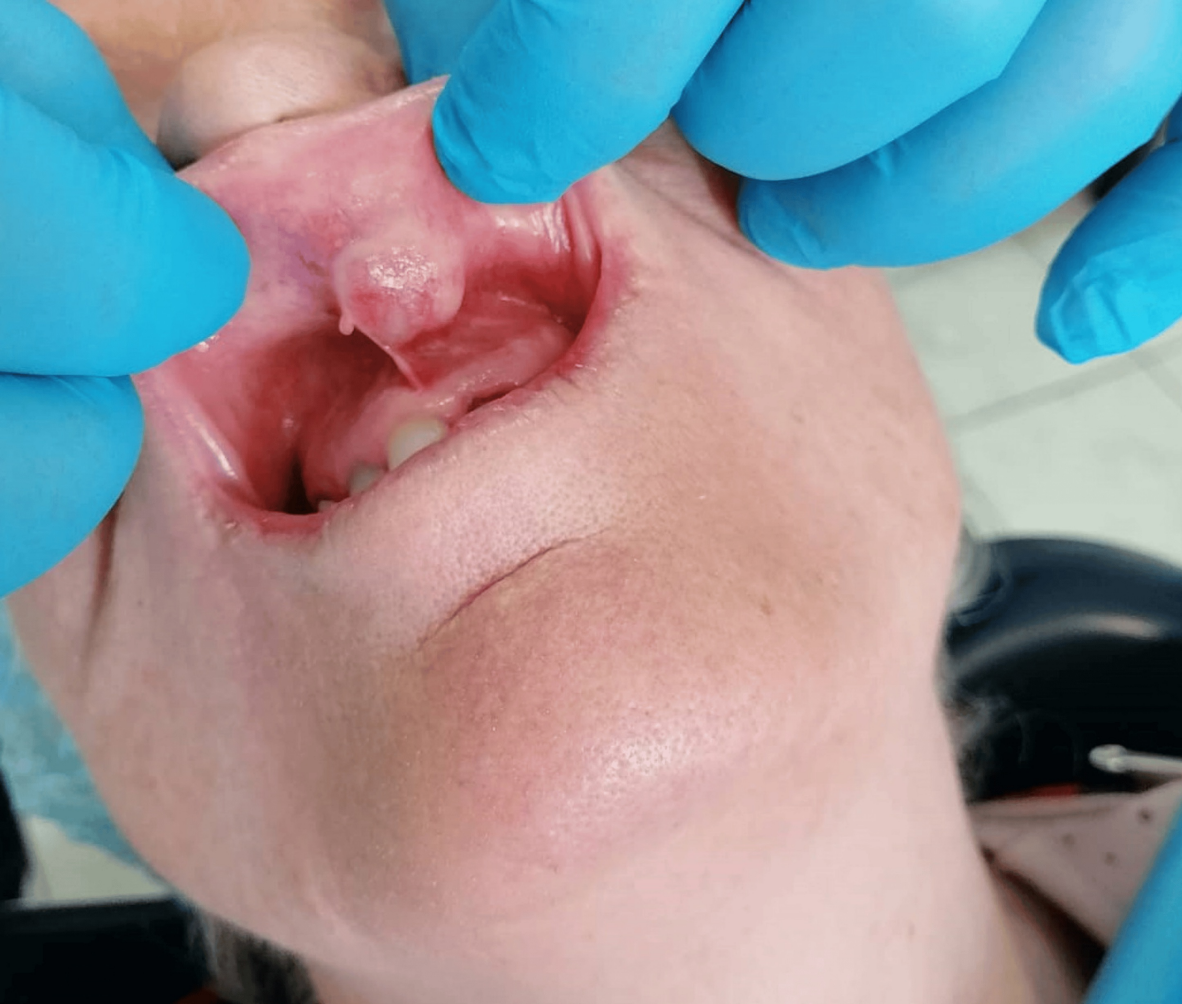
The initial presentation of a single lobulated, normal-coloured, 10 mm mass on the upper lip

We present the case of a 68-year-old female patient who was referred to our hospital (University General Hospital of Patras, Greece) due to a 10-year history of a mass located in the middle portion of the upper lip. Clinically, the lesion was hard, painless and mobile on palpation, with stable dimensions over the years, measuring approximately 10 mm in greatest diameter (Figure 1).

**Figure 2:**
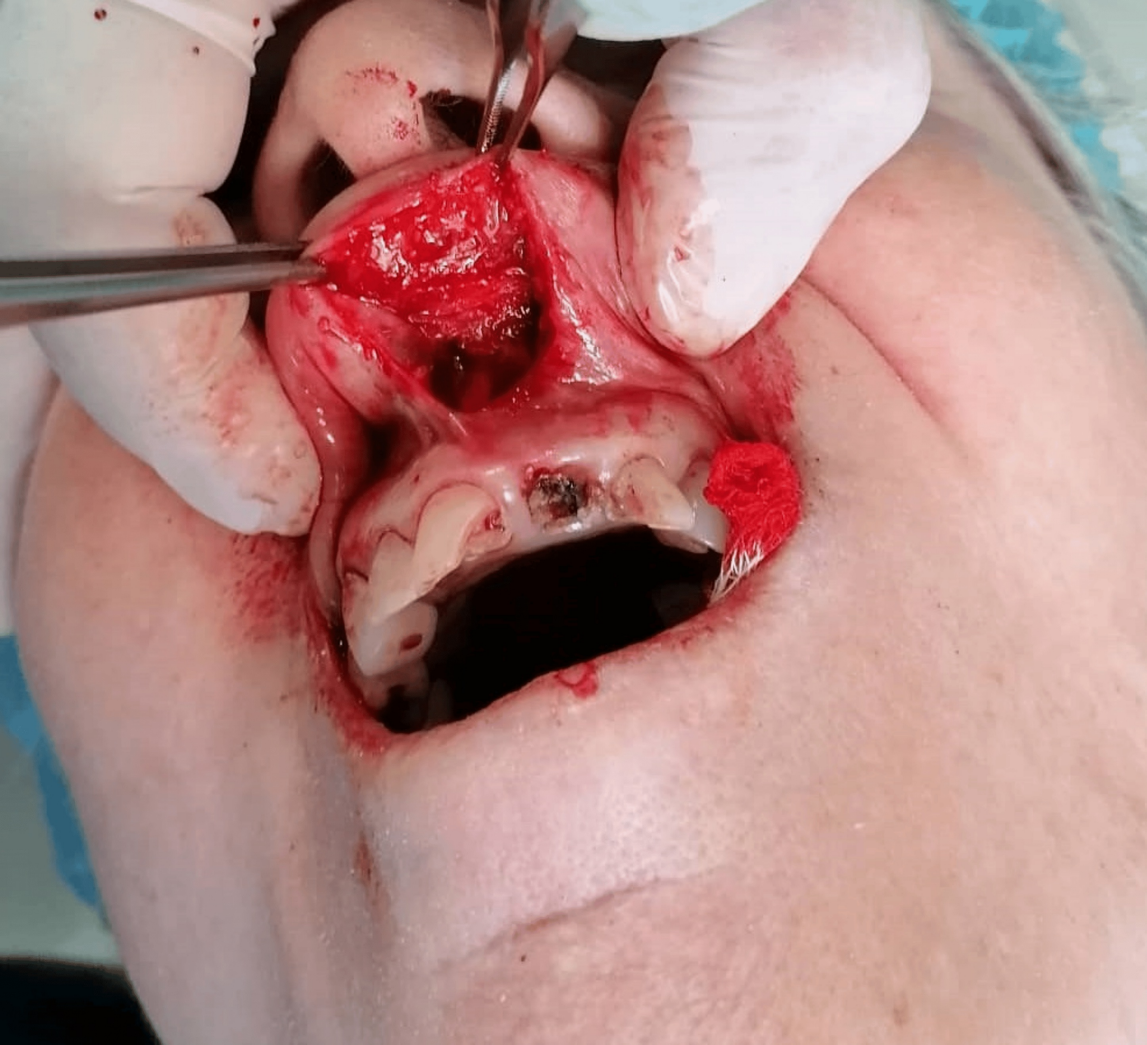
Post-surgical appearance, after total excision of the canalicular adenoma

Regarding morphology, the lesion was covered by normal-coloured mucosa and showed no signs of inflammation. No swollen cervical lymph nodes were detected. The patient’s medical history was significant for hypertension and hyperlipidaemia, with no previous radiation exposure. In terms of medications, she was on olmesartan and rosuvastatin and had no known drug allergies. The initial differential diagnosis included a benign oral neoplasm and chronic sialadenitis of the minor salivary glands. The lesion was surgically removed under local anaesthesia (Figure 2).

**Figure 3:**
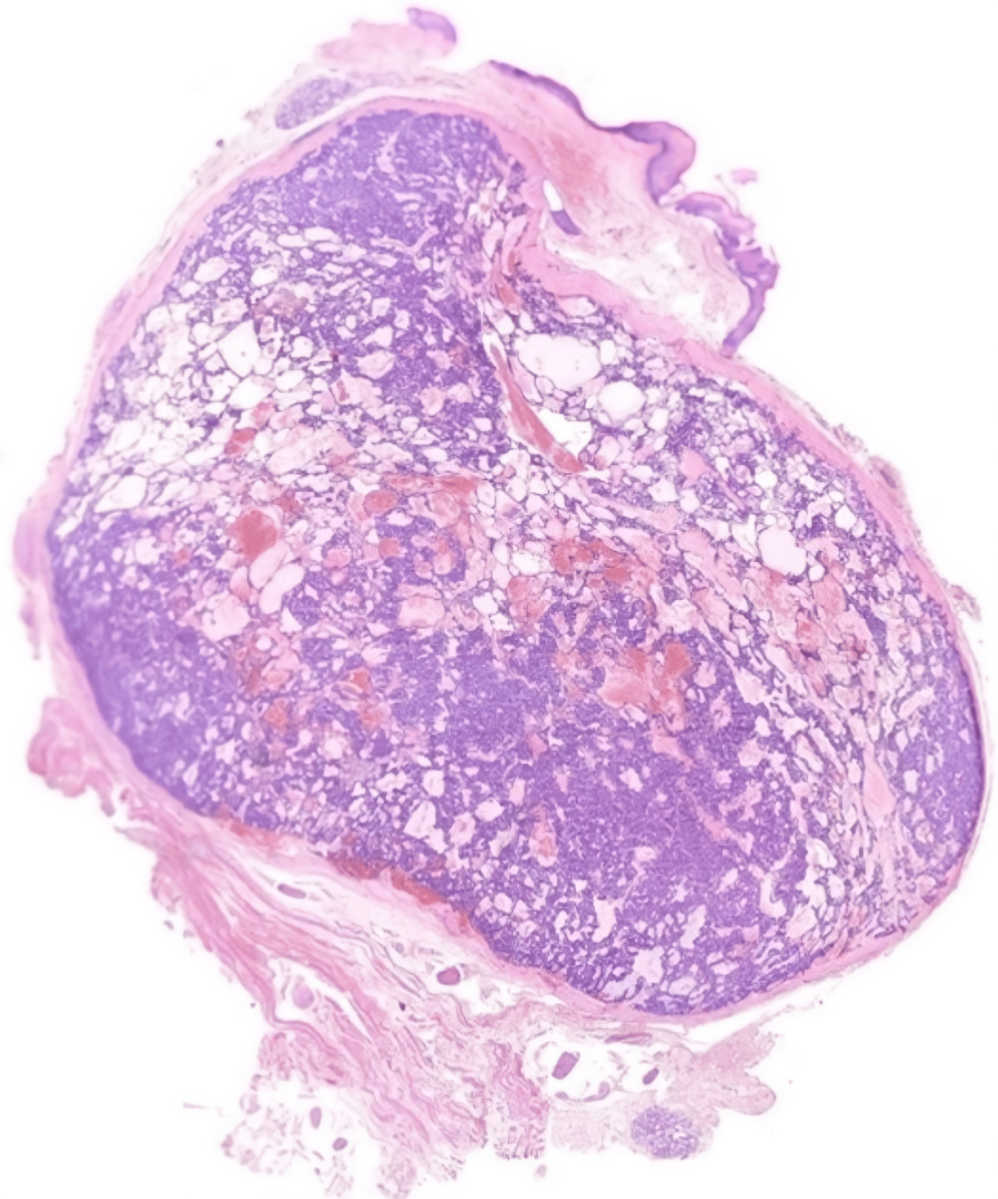
Microscopically, the tumour was submucosal, well-circumscribed and encapsulated. Cellular and cystic areas are assessed at low power (H-E, 0.5x).

**Figure 4:**
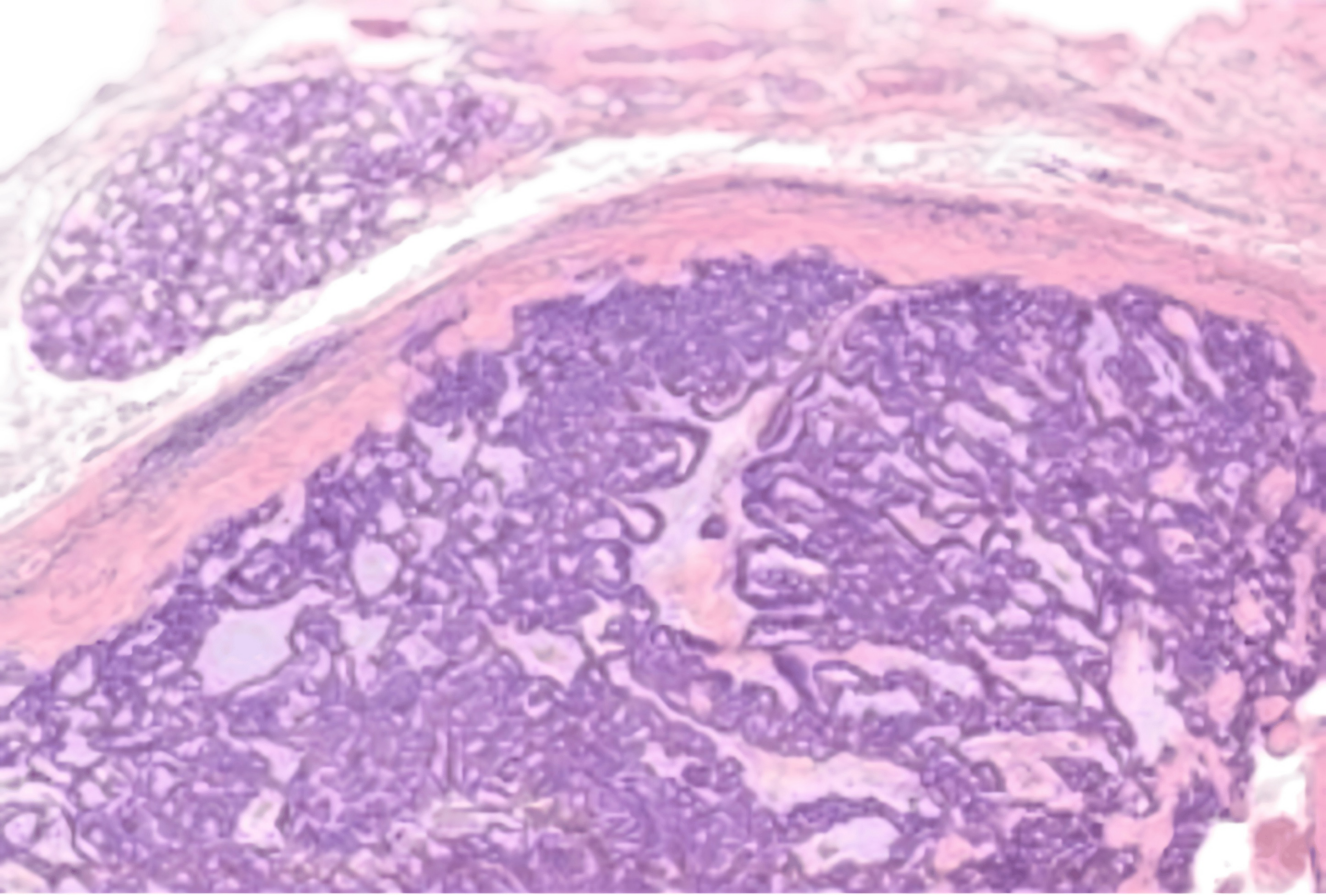
The neoplasm is composed of anastomosing cords forming tubular structures with a myxoid background. Normal salivary gland tissue is seen at the periphery (upper left corner) (H-E, 10x).

**Figure 5:**
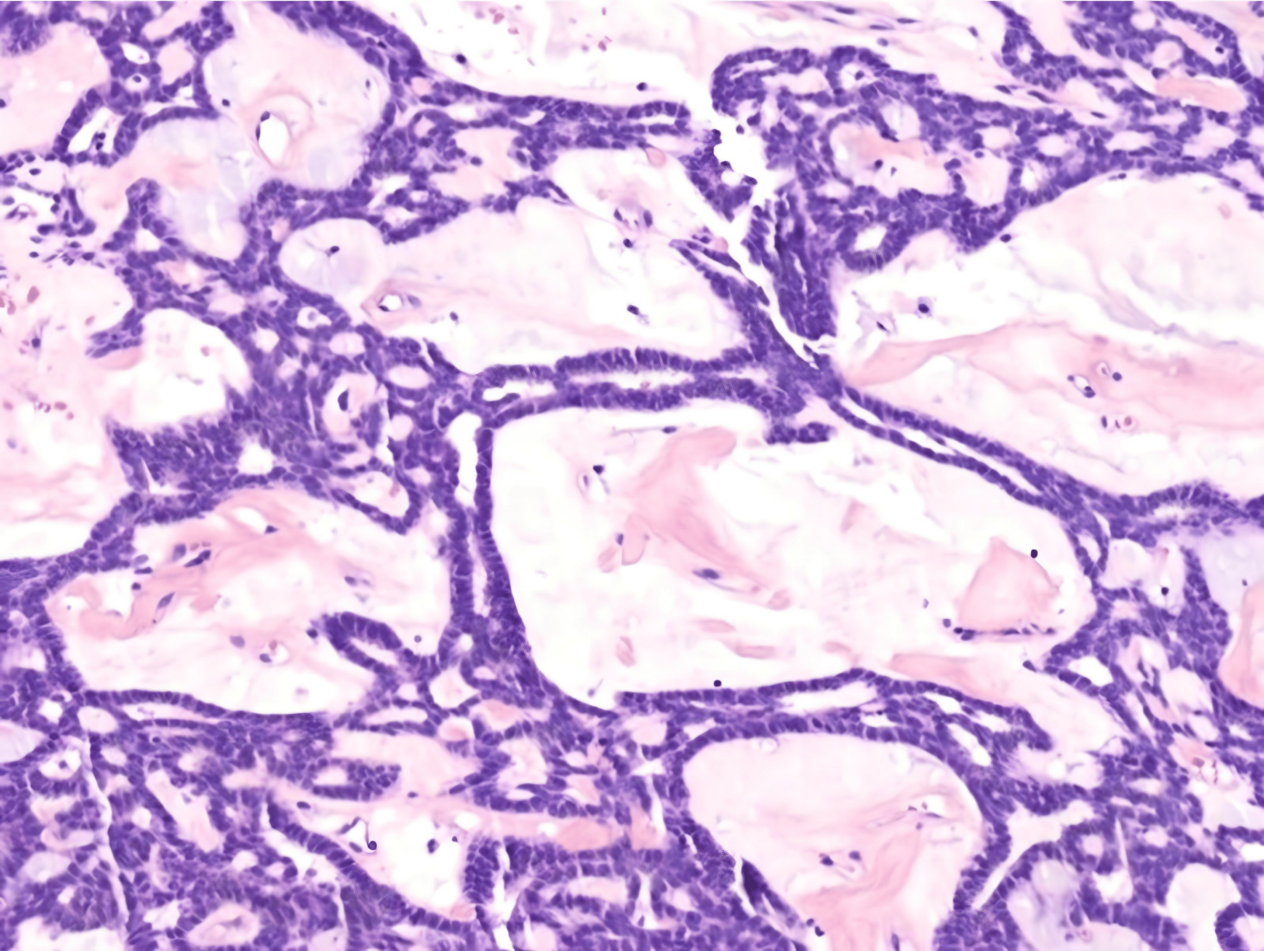
Tumour cells are monomorphic and exhibit a unique “beading” pattern of CA, with parallel rows of cells that focally interconnect (H-E, 20x). CA: Canalicular adenoma

**Figure 6:**
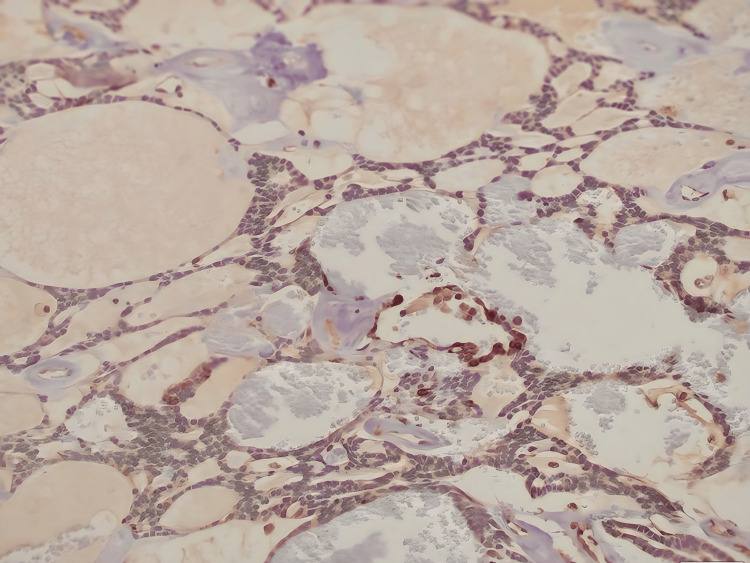
GFAP has focally a linear expression pattern around the lumina of the cystic structure (GFAP IHC, 20X). GFAP: Glial fibrillary acidic protein

On gross examination, the tumour was well-circumscribed, with a firm and gelatinous tan cut surface. Histological examination revealed a submucosal nodule surrounded by a fibrous capsule (Figure 3). The tumour showed a lattice-like pattern of anastomosing cords and strands of cells with tubule and cyst formation (Figure 4). Areas of tight glandular architecture with a syncytial appearance of cells were noticed. The frequently described, characteristic “beading” pattern, consisting of parallel rows of cells merging together, was also present (Figure 3). The neoplasm consisted of a monotonous population of cuboidal to columnar cells with ovoid nuclei, fine chromatin, only focal, small nucleoli and moderate to scant amount of eosinophilic cytoplasm (Figure 5). Focal pseudostratification of nuclei and rare mitoses were noted; however, atypical mitoses were absent. The surrounding stroma was oedematous, with myxoid and sclerosing areas. Intraluminal haemorrhage inside the cystic spaces was noted. The neoplasm was completely excised, and normal salivary gland lobules were observed at the periphery (Figure 6). The overlying epithelium of the oral mucosa showed mild hyperplasia and focal parakeratosis. Immunohistochemically, the tumour cells expressed epithelial markers CK8/18 and CK7, as well as S100 and c-kit. Glial fibrillary acidic protein (GFAP) was focally positive, especially along the luminal border of the cells (Figure 4). The myoepithelial marker SMA and markers CEA, p63 and BCL-2 were negative. Beta-catenin showed membranous expression. Following the surgical excision, the patient was monitored for a few hours, prescribed a broad-spectrum antibiotic, and subsequently discharged from the hospital. No recurrence occurred during the two-year follow-up.

## Discussion

Clinical presentation

Salivary gland tumours are relatively rare, accounting for only 3% of head and neck neoplasms [6]. CAs are uncommon tumours of the minor salivary glands that can arise throughout the oral cavity. Of note, the upper lip is the main presentation site of CAs, accounting for approximately 68-81% of cases, while the buccal mucosa and palate represent 16% and 10% of cases, respectively [2]. In addition, this entity constitutes 20% of benign tumours of the upper lip and rarely occurs in the major salivary glands (e.g. parotid gland) and oesophagus [2,6,7]. Patients are typically older than 50 years of age [2] and women are nearly twice as likely to be affected as men [4]. The lesion is typically an asymptomatic, gradually growing mass, with single or multinodular localisation in the oral cavity [3]. The differential diagnosis includes both benign and malignant salivary gland tumours, mucocele, and sialadenitis due to sialolithiasis and oral mucosa lesions (e.g. lipoma, fibroma, pyogenic granuloma). Definitive diagnosis requires histologic assessment.

Histology and immunohistochemistry

CAs have distinctive histologic features. They have a bosselated or multilobulated circumference and are well-delineated tumours, often with a fibrous capsule. Their growth pattern is that of double columns of monomorphic cells in anastomosing cords, tubules, canals, strands and/or cysts [8]. The “beading” pattern is unique to canalicular adenomas, referring to the parallel, separated rows of cells that appear to focally merge like knots [8,9]. Papillary projections and intraluminal, squamoid morules may also be observed. The cells are uniform, with round to oval pseudostratified nuclei, coarse chromatin, moderate to scant amount of eosinophilic cytoplasm, and indistinct cell borders. Mitoses are rare, and importantly, CAs lack myoepithelial cells. The neoplastic cells are set in loose, myxoid or hyalinised stroma with rich vasculature. Microliths are occasionally noticed [8,10]. Immunohistochemically, CA cells exhibit reactivity for pan-cytokeratin and S-100 [11]. GFAP stains in a characteristic linear fashion along the luminal surface or at the interface between the cells and stroma [8,12]. C-KIT, vimentin and SOX10 expression have also been described [8,11,13]. In approximately 75% of CAs, p63 shows cytoplasmic expression, while nuclear expression of p63 as well as p16 (not associated with high-risk HPV) is found in the squamous morules [8]. P63 was negative in our case. DOG-1 and myoepithelial markers are negative [14,15]. The histological differential diagnosis of CAs includes both benign and malignant salivary gland tumours. Benign tumours that should be considered in the differential diagnosis include basal cell adenoma, pleomorphic adenoma, striated duct adenoma and myoepithelioma. More importantly, CAs need to be distinguished from malignant salivary gland tumours, namely polymorphous adenocarcinoma and adenoid cystic carcinoma.

Histological differential diagnosis

The histological differential diagnosis of CAs includes both benign and malignant salivary gland tumours. CAs may resemble another monomorphic benign salivary gland tumour, basal cell adenoma. As previously mentioned, according to the nomenclature of the past, CAs were considered a subtype of basal cell adenoma; however, some distinguishing features enabled their separation in the latest WHO editions [8]. In contrast to CAs, the vast majority of basal cell adenomas arise in major salivary glands and mainly in the parotic gland. Basal cell adenoma is also well-circumscribed and composed of uniform cells forming nests and trabeculae with frequent peripheral palisading. In the tubulotrabecular growth pattern, branching duct-like structures may mimic the interconnecting canals of CAs; however, cells do not appear to align in columns. The stroma also usually differs having a more fibrocellular nature in basal cell adenoma as opposed to the more hypocellular and loose stroma of CAs [9,16,17]. A spindled component and basal membrane material may be present in basal cell adenoma [16]. This tumour is composed of a dual cellular population with myoepithelial cells present, which can be confirmed with myoepithelial markers [15]. Nuclear beta-catenin is also described [18]. In our case, beta-catenin was expressed only in a membranous pattern. Positivity for BCL-2 and CEA is found in a minority of cases [8]. Pleomorphic adenoma has also a biphasic population of epithelial and myoepithelial cells that helps differentiate from CAs; however, maybe a more readily seen feature is the chondroid or chondromyxoid stroma, which is absent in CAs [8]. Striated duct adenoma is another benign, rare salivary gland tumour that predominates in the parotid, is composed of closely packed ducts and is easily distinguished from CAs as it essentially lacks intervening stroma [8]. In myoepithelioma, neoplastic cells may have epithelioid morphology and may be arranged in anastomosing trabeculae; however, the lack of specific features of CAs (e.g., “beading”) and the diffuse positivity for myoepithelial markers guide the diagnosis [8,19]. More importantly, CAs need to be distinguished from malignant salivary gland tumours. Polymorphous adenocarcinoma may appear as a well-circumscribed but unencapsulated tumour. This tumour should be considered in the differential diagnosis due to its cytologic uniformity and some shared architectural patterns with CAs, among the many polymorphous adenocarcinoma. However, cells in polymorphous adenocarcinoma show open chromatin. Perineural invasion as well as an infiltrative growth are helpful in the diagnosis [8]. Adenoid cystic carcinoma can be distinguished from CAs due to its dual cellular population, cribriform architecture, prominent perineural invasion and infiltrative growth (Table 1) [8].

**Table 1:** Histological differential diagnosis and histopathological features

Tumour	Histopathological features
Canalicular adenoma	Bosselated or multilobulated, well-delineated tumour often with a fibrous capsule double columns of monomorphic cells in anastomosing cords, tubules, canals, strands and/or cysts “beading” pattern (unique to canalicular adenoma) +/- papillary projections and intraluminal squamoid morules uniform cells with pseudostratified nuclei, coarse chromatin, eosinophilic cytoplasm, and indistinct cell borders rare mitoses lack of myoepithelial cells stroma: hypocellular and loose, myxoid or hyalinized with rich vasculature
Basal cell adenoma	Uniform cells forming nests and trabeculae with frequent peripheral palisading biphasic population of epithelial and myoepithelial cells fibrocellular stroma +/- spindled stromal component and basal membrane material nuclear beta-catenin
Pleomorphic adenoma	Biphasic population of epithelial and myoepithelial cells chondroid or chondromyxoid stroma
Striated duct adenoma	Closely packed ducts with minimal intervening stroma
Myoepithelioma	Variable morphology including epithelioid appearance variable architectural patterns including trabecular pattern diffuse positivity for myoepithelial markers
Polymorphous adenocarcinoma	Nuclei with open chromatin perineural invasion infiltrative growth
Adenoid cystic carcinoma	Dual cellular population cribriform architecture prominent perineural invasion infiltrative growth

Origin

Based on the immunophenotypic as well as the ultrastructural characteristics of CA cells, attempts have been made to identify the cell of origin, with conflicting results. Some suggest an origin from the epithelial cells of the intercalated, striated, or excretory ducts of the salivary glands [2]. Another possibility is the origin from a pluripotent basal reserve cell [2,20]. However, evidence suggests that the phenotype of CAs may be unrelated to their histogenesis and instead result from functional alterations due to factors in the microenvironment [10]. 

Prognosis

The prognosis is generally good, with recurrences being rare, occurring in only 5% of cases [3]. However, CAs require long follow-up, as they are often multifocal and can lead to delayed recurrence. It is extremely important to remove all localised foci during the initial presentation. Notably, CAs do not exhibit malignant transformation, and each new occurrence is not a sign of malignancy [2].

## Conclusions

CAs are uncommon benign salivary gland tumours with a predilection for elderly women. These tumours should be considered in the clinical evaluation of a painless mass of the upper lip. Although their clinical features are not typically suspicious for malignancy, surgical resection and histologic examination are necessary for diagnosis and exclusion of malignancy. CAs have a distinct morphology and unique, diagnostically helpful, histological features. Complete surgical excision is curative, and recurrence rates are low.
